# Microinjection of pruritogens in NGF-sensitized human skin

**DOI:** 10.1038/s41598-021-00935-x

**Published:** 2021-11-02

**Authors:** Hans Jürgen Solinski, Roman Rukwied, Martin Schmelz

**Affiliations:** Department of Experimental Pain Research, MCTN, Medical Faculty Mannheim, Heidelberg University, Theodor-Kutzer-Ufer 1-3, 68167 Mannheim, Germany

**Keywords:** Neurotrophic factors, Peripheral nervous system, Sensory processing, Somatosensory system, Neurophysiology

## Abstract

Single intradermal injections of nerve growth factor (NGF) evoke prolonged but temporally distinct sensitization patterns to somatosensory stimuli. Focal administration of the non-histaminergic pruritogen cowhage but not histamine resulted in elevated itch at day 21 after NGF administration. Here, we injected bovine adrenal medulla peptide 8–22 (BAM8–22), β-alanine (β-ALA) and endothelin-1 (ET-1) into NGF-treated skin of 11 healthy volunteers and investigated the corresponding itch/pain and flare reactions. β-ALA was the weakest pruritogen, while BAM8–22 and ET-1 were equally potent as histamine. NGF did not sensitize itch or flare reactions induced by any compound, but injection and evoked pain were increased at day 21 and 49. The involvement of histamine H1 receptors in itch was explored in eight subjects after oral cetirizine. ET-1-induced itch and flare were significantly reduced. BAM8–22 and β-ALA itch were not affected, but flare responses after BAM8–22 reduced by 50%. The results indicate that a single NGF injection does not sensitize for experimentally induced itch but increases pain upon pruritogen injection. In healthy humans, pruritic and algetic processing appear differentially regulated by NGF. However, in patients suffering chronic itch, prolonged elevation of NGF-levels under inflammatory conditions may contribute to elevated itch.

## Introduction

Itch is defined as an unpleasant skin sensation and is associated with the strong urge to scratch. It can be induced directly, e.g. by mechanical or chemical stimulation, or indirectly, e.g. upon skin mast cell degranulation accompanied by histamine (HIS) release. The best-studied pruritogen, HIS, induces itch and a concurrent flare reaction in humans by activation of a specific subset of mechanically insensitive C-fibers (CMi^1,2^). Other pruritogens, collectively referred to as non-histaminergic, use divergent yet incompletely defined neuronal pathways to induce itch^3^. Among these, bovine adrenal medulla peptide 8–22 (BAM8–22), β-alanine (β-ALA) and endothelin-1 (ET-1) have been shown to induce itch in healthy volunteers upon intradermal microinjections^4–6^. Their cognate receptor proteins—MRGPRX1, MRGPRD and ENDRA—are expressed in human dorsal root ganglion (DRG) neurons in a largely overlapping fashion^7–9^, suggesting a direct stimulatory effect of these pruritogens on pruritic primary afferents. These neurons also express transduction proteins normally linked to painful stimulation, e.g. the transient receptor potential cation channel subfamily V member 1 (TRPV1)^8,9^. In contrast to intradermal microinjection of the TRPV1 agonist capsaicin, which causes pain, focal capsaicin administration induces itch of similar intensity as histamine^10^ or BAM8–22^5^. Specific discharge patterns evoked by either pruritic or algetic stimuli, particular neuronal population codes or a combination of both are currently the favored models to explain how exactly non-histaminergic itch is encoded in humans and can be differentiated from pain^11^.

In the context of chronic itch, anti-histamines are largely ineffective, pointing to non-histaminergic mechanisms as dominating drivers in most chronic itch entities. The group of LaMotte developed an experimental model in healthy volunteers that recapitulated many features of chronic itch patients, including spontaneous itch that persisted for about 1 week, increased pruritogen-induced itch, hyperknesis and alloknesis^12^. Interestingly, mechanical and heat hyperalgesia were also observed in this model, suggesting that sensitization pathways to pruritogens and algogens might partially overlap.

The neurotrophic protein nerve growth factor (NGF) has such a dual role, being implicated in sensitization to both, itch and pain. Mechanistically, after binding of NGF to the high-affinity NGF receptor neurotrophic receptor tyrosine kinase 1 (NTRK1), the NGF-NTRK1-receptor complex is internalized and transported along the axon to the DRGs resulting in an increased de novo protein biosynthesis^13^. The retrograde transport of newly synthetized receptor proteins and ion channels (for instance TRPV1) to the peripheral sensory endings have been assumed as molecular mechanisms for NGF-induced hyperalgesia^14–16^. In addition, local axonal protein synthesis might contribute to the previously observed long-lasting NGF-evoked sensitization confined to the NGF injection site^17–19^. Clinically, elevated tissue NGF levels or expression patterns were associated with chronic inflammatory pain, such as osteoarthritis^20^ or pancreatitis^21^. Enhanced peripheral neuronal sprouting and a correspondingly increased density of sensory endings induced by NGF were suggested to contribute to an increased nociceptive input and pain, as shown for instance in disease models of bone cancer pain^22,23^. Thus, high NGF levels in chronic pain patients have been associated with facilitated nociception under inflammatory conditions and intriguingly, monoclonal antibodies targeting NGF substantially ameliorated their pain^24,25^. These reports establish a crucial role of NGF for chronic inflammatory pain, whereas the role of NGF for chronic inflammatory itch remains less defined: In atopic dermatitis (AD), psoriasis or contact dermatitis, elevated NGF-levels in the horny layer of the epidermis were found to positively correlate with itch intensity^26,27^. Moreover, chronic itch in psoriasis patients could be reduced significantly by topical treatment with a NTRK1-inhibitor^28^ and it will be of interest to pursue anti-NGF therapies targeting itch in AD patients.

In order to specifically test whether the elevated NGF-levels found in clinical inflammatory itch conditions are sufficient to facilitate itch in humans, we sensitized skin nociceptors in healthy subjects with NGF and assessed pruritogen-induced sensations. A single intracutaneous injection of NGF induces a mechanistically specific and several weeks lasting hyperalgesia in volunteers. In contrast to facilitated pain, we demonstrated previously that histaminergic itch was not significantly enhanced in NGF-sensitized skin^29,30^, whereas cowhage-evoked non-histaminergic itch was increased at the time of maximal mechanical hyperalgesia (about day 21) but unchanged at maximum heat hyperalgesia (about day 3) after NGF administration^29,30^. Considering that non-histaminergic pruritus plays a significant role in clinical inflammatory itch^31^, we tested for sensitized itch and pain after experimental activation of the NGF-NTRK1 signaling pathway in human skin. At the time of maximum heat and mechanical hyperalgesia, we administered the well-established human pruritogens BAM8–22, β-ALA, and ET-1, and assessed the corresponding itch and pain response with HIS serving as pruritic control stimulus.

## Results

### BAM8–22, β-ALA, and ET-1 induced itch and flare responses

**Figure 1:**
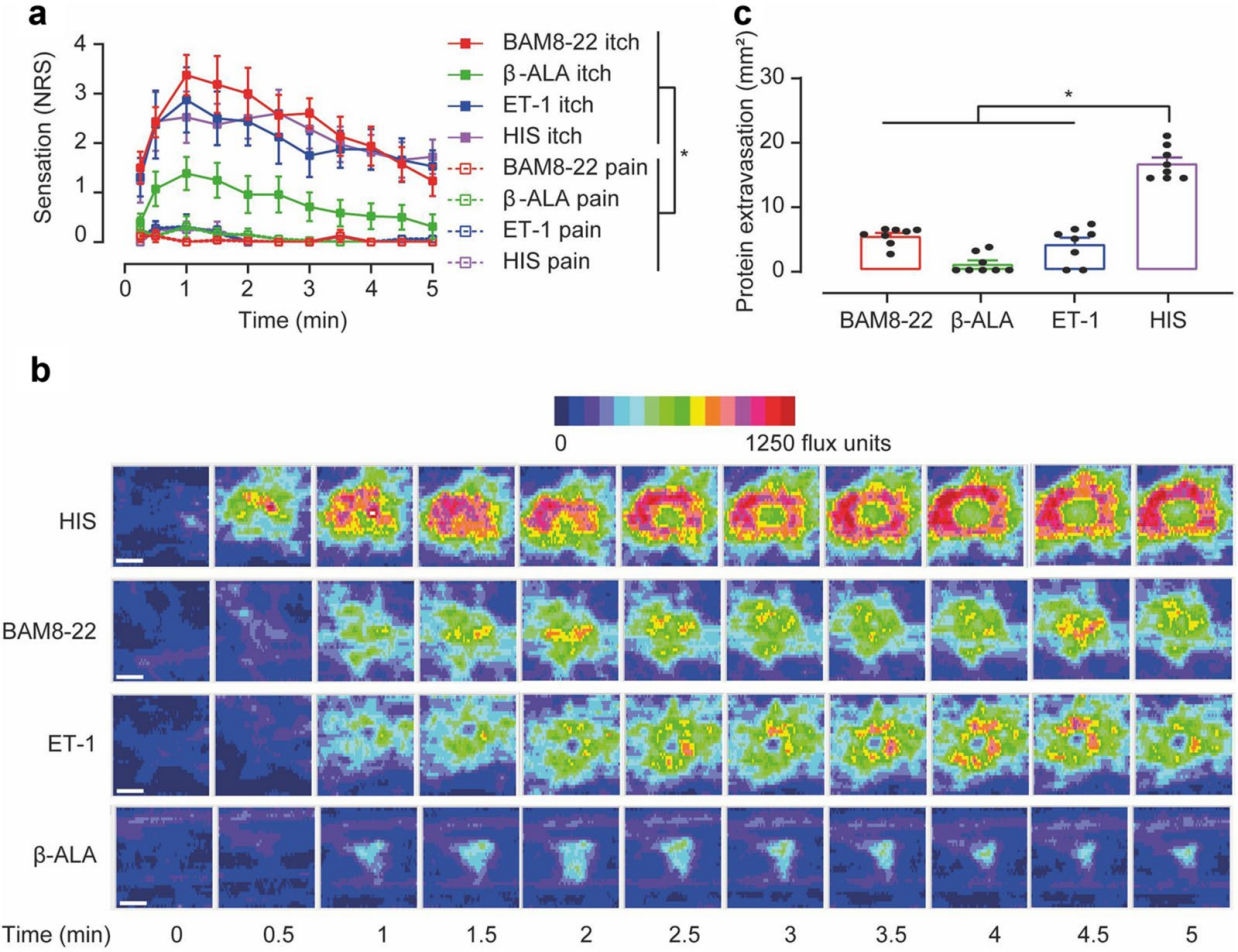
BAM8–22, β-ALA, and ET-1 induce itch in humans and cause differing degrees of flare responses. (**a**) Itch (solid symbols) and pain ratings (open symbols) recorded on a numeric rating scale (NRS, 0–10) over 5 min post pruritogen delivery (n = 8). For all substances, itch was the dominant sensation (all p < 0.05, marked with asterisk). Data are shown as mean ± standard error (SEM). (**b**) Specimen of a laser Doppler imaging sequence recorded after HIS, BAM8–22, ET-1 and β-ALA delivery (from top to bottom) in 0.5 min intervals for 5 min. Color changes (from blue to red) indicate increase of skin blood flow (flux units), the scale bar indicates 1 cm. (**c**) Protein extravasation (mm^2^) assessed 5 min after pruritogen delivery. Note that HIS induced a substantially larger area of protein extravasation compared to BAM8–22/β-ALA/ET-1 (all p < 0.0001, marked with asterisk). Data are shown as mean ± standard error (SEM) with overlay of individual data points.

We first studied the psychophysical and vascular effects to intradermal microinjections of BAM8–22, β-ALA and ET-1 in healthy human volunteers and compared the results to a HIS iontophoresis delivered as pruriceptive control stimulus. In general, itch sensations developed with a rapid onset that peaked about one minute after compound administration and declined thereafter over 5 min (Fig. 1a). While BAM8–22, ET-1 and HIS induced strong itch with peak intensities of ~ 3 on the NRS scale, β-ALA induced a weaker itch sensation with about half the maximum intensity.

Although itch was the dominating sensation, all chemicals also caused weak pain sensations of NRS < 1 (Fig. 1a). Calculating the area under the curve (AUC) for both, itch and pain, revealed that each pruritogen induced significantly more itch than pain (BAM8–22, ET-1, HIS: p < 0.0001; β-ALA: p < 0.05).

Increased skin blood flow around the pruritogen administration sites (axon-reflex flare) was measured by laser Doppler imaging (LDI). BAM8–22 and ET-1, but not β-ALA, induced significant flare reactions (specimen in Fig. 1b). The size and time-course of this response were similar to those induced by HIS iontophoresis.

Extravasation of plasma proteins (wheal response) due to an increased permeability of post-capillary venules was planimetrically assessed. Iontophoresis of HIS evoked a pronounced protein extravasation in addition to the flare response. In contrast, injections of BAM8–22 and ET-1 caused only a minute protein extravasation and β-ALA did not produce a wheal at all, arguing against a major HIS release by these three pruritogens (Fig. 1c, HIS vs. BAM8–22/β-ALA/ET-1 p < 0.0001).

**Figure 2:**
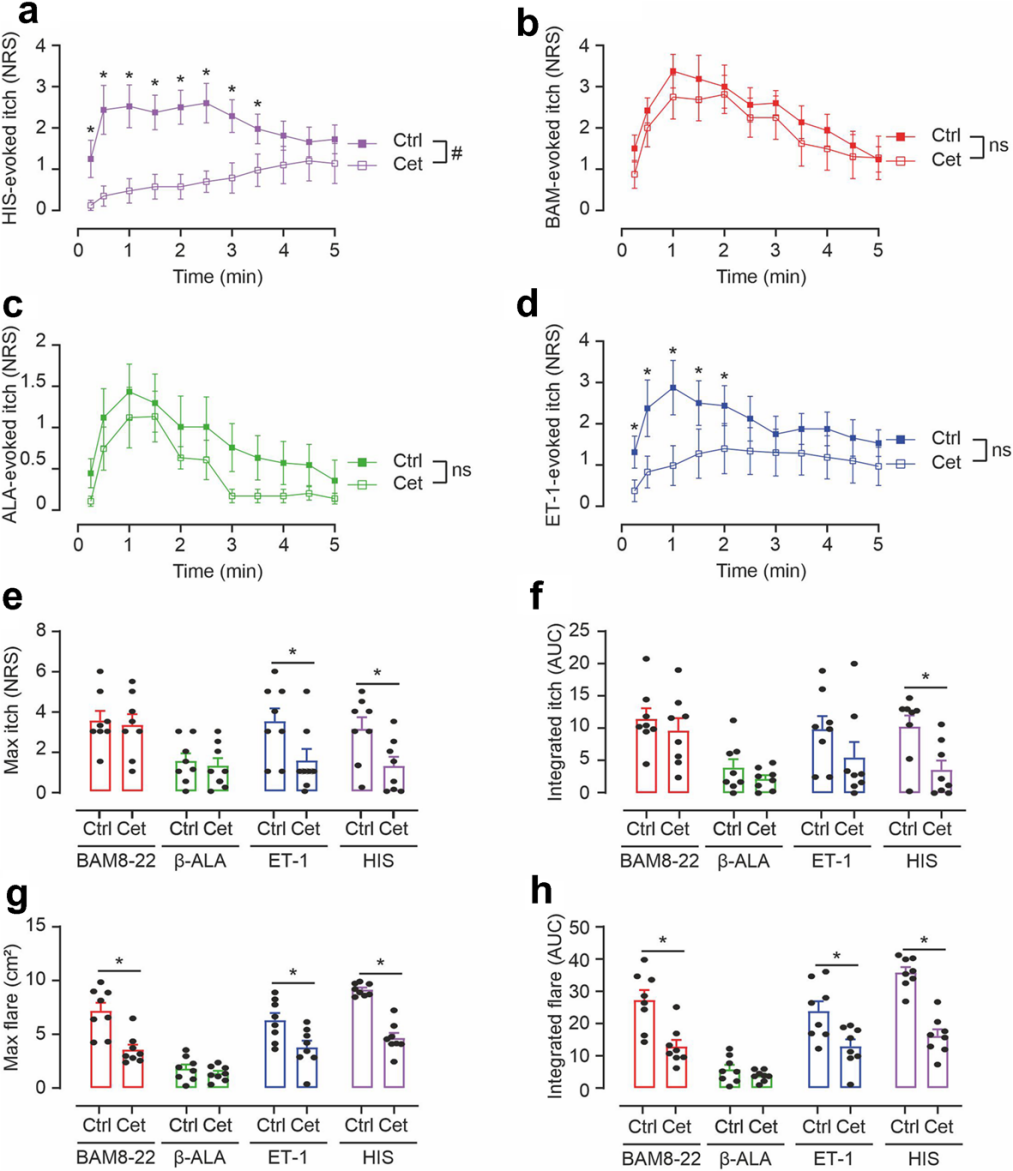
BAM8–22, β-ALA, and ET-1 evoke somatosensory and vascular responses via HIS-dependent and HIS-independent mechanisms. (**a–d**) Itch recorded on a numeric rating scale (NRS, 0–10) for 5 min after pruritogen delivery in 8 subjects before (Ctrl, solid symbols) and 3 h after oral HRH1-antagonist cetirizine (Cet, open symbols). Cet treatment diminished ET-1-evoked itch at early time-points (p < 0.05, marked with asterisk), whereas HIS-induced itch was decreased overall (p < 0.01, marked with hash sign) and in particular at early time-points during the 5-min observation period (p < 0.05, marked with asterisks). Note that Cet had no effect on BAM8–22- and β-ALA-evoked itch (n.s.). Data are shown as mean ± standard error (SEM). (**e,f**) Group comparison of maximum itch (NRS) and integrated itch (AUC) evoked by the pruritogens before (Ctrl) and 3 h after cetirizine (Cet). Maximum itch upon ET-1 and HIS as well as integrated itch after HIS delivery were significantly reduced by Cet (p < 0.02, marked with asterisks) whereas responses to BAM8–22 and β-ALA were not affected. Data are shown as mean ± standard error (SEM) with overlay of individual data points. (**g,h**) Maximum flare (cm^2^) and integrated flare (AUC) recorded during 5 min after pruritogen administration before (Ctrl) and after cetirizine (Cet). Maximum and integrated flare responses upon BAM8–22, ET-1, and HIS challenge were significantly attenuated by Cet (all p < 0.0001, marked with asterisks). Data are shown as mean ± standard error (SEM) with overlay of individual data points.

Nonetheless, to check for an indirect effect of the non-histaminergic pruritogens via the release of HIS from e.g. skin mast cells, we pre-treated our subjects with the anti-histamine Cetirizine (Cet) 3 h prior to pruritogen challenge. As expected, the anti-histamine significantly reduced the HIS-induced itch, flare and protein extravasation (Fig. 2a+e–h, Fig. S1A). BAM8–22- and β-ALA-induced itch sensations (time-course, maximum itch and integrated itch assessed by calculating the area under the curve AUC)) were not affected by Cet-pretreatment, while ET-1-induced itch was significantly diminished (Fig. 2b–f). Despite the significant reduction of peak itch intensity of ET-1 from NRS 3.5 to 1.5 (Fig. 2e, p < 0.02), a low-intensity itch was still present throughout the observation time. Accordingly, integrated itch (AUC) of ET-1 was not significantly different after Cet-pretreatment (Fig. 2f, n.s.).

Cet also potently inhibited the ET-1-induced flare response (Fig. 2g+h, p < 0.0001) and surprisingly, BAM8–22-induced flare was also inhibited significantly by ~ 50% (Fig. 2g+h, p < 0.0001). The small protein extravasation (Fig. S1A) and mild pain induced by pruritogen microinjections (Fig. S1B+C) were unaffected by the anti-histamine.

### BAM8–22-, β-ALA-, and ET-1-induced responses in NGF-sensitized skin

We assessed sensory and vascular changes induced by BAM8–22, β-ALA, ET-1, and HIS at day 3, day 21 and day 49 after recombinant human NGF (rhNGF) injection. At these time-points we expected maximal heat hyperalgesia (day 3), maximal mechanical hyperalgesia (day 21) and a largely resolved sensitization (day 49).

Injection pain was rated on a numeric rating scale (NRS, 0–10) for rhNGF and saline control (day 0). Volunteers reported a mild burning pain sensation of NRS 1.9 ± 0.2 upon rhNGF injection that was not significantly different from saline injection (NRS 1.3 ± 0.2, n.s.). Neither rhNGF- nor saline-injection induced visible erythema and wheal reactions in the skin or any non-evoked, ongoing sensation. Injection of BAM8–22, β-ALA, and ET-1 into the pre-treated skin sites caused stronger pain in rhNGF-sensitized compared to saline control skin (main effect “rhNGF vs. saline” for BAM8–22 (p < 0.005) and ET-1 (p < 0.02). Increased injection pain upon β-ALA administration was only detected at day 21 post rhNGF injection and was significantly higher as compared to ET-1 (p < 0.005, Bonferroni post-hoc test) but not to BAM8–22 (n.s., Table 1).

**Table 1:** rhNGF enhances pain during injections of BAM8–22, β-ALA and ET-1.

	BAM8–22		β-ALA		ET-1	
	Saline	NGF	Saline	NGF	Saline	NGF
Day 3	0.34 ± 0.20	1.14 ± 0.26	1.96 ± 0.51	1.73 ± 0.54	0.55 ± 0.17	0.77 ± 0.29
Day 21	0.61 ± 0.22	1.83 ± 0.42*	1.39 ± 0.49	3.06 ± 0.58*	0.50 ± 0.20	0.94 ± 0.36
Day 49	0.82 ± 0.25	1.23 ± 0.39	0.78 ± 0.33	0.56 ± 0.24	0.34 ± 0.20	1.46 ± 0.35*

Maximum pain recorded on a numeric rating scale (NRS, 0–10) in response to injections of BAM8–22, β-ALA, and ET-1 at 3 days (n = 11), 21 days (n = 9) and 49 days (n = 11; n = 8 for β-ALA) post rhNGF and saline administration, respectively.

Values are given as mean ± standard error (SEM), asterisks indicate significant differences between rhNGF and saline (p < 0.02).

**Figure 3:**
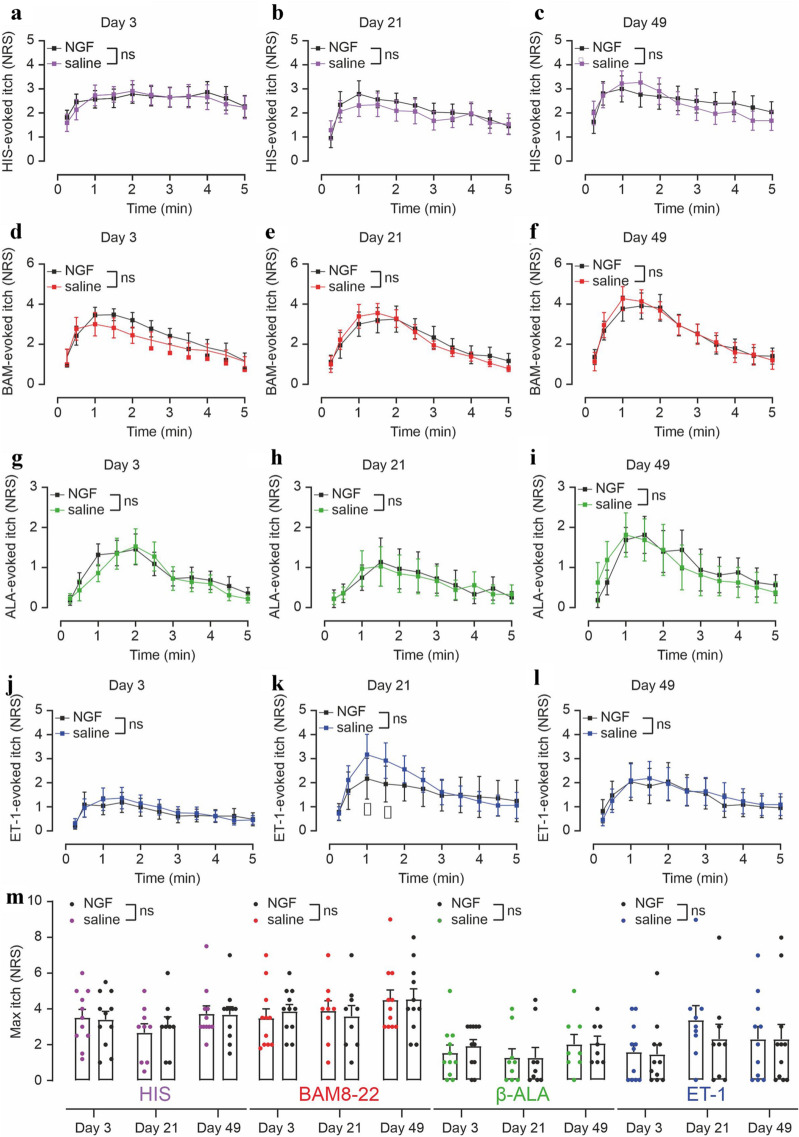
rhNGF does not sensitize itch to BAM8–22, β-ALA and ET-1. (**a–l**): Itch ratings (NRS, 0–10) upon HIS (**a–c**), BAM8–22 (**d–f**), β-ALA (**g–i**), and ET-1 (**j–l**) recorded for 5 min at 3 days (n = 11), 21 days (n = 9) and 49 days (n = 11; n = 8 for β-ALA) post injection of rhNGF (black symbols) and saline (colored symbols), respectively. Overall, significant differences between the rhNGF- and saline-treated sites were not detected (n.s.). Asterisks indicate significant interaction “rhNGF vs saline” × “time” after ET-1 injection (p < 0.001). Data are depicted as mean ± standard error (SEM). (**M**) Maximum itch recorded during the 5-min observation period did not indicate a significant difference between rhNGF and saline at any day of the observation (n.s.). Data presented as mean ± standard error (SEM) with overlay of individual data points.

Histaminergic itch was neither enhanced nor diminished by rhNGF treatment on any of the three experimental days (Fig. 3a–c,m). Likewise, BAM8–22-, β-ALA-, and ET-1-induced itch were unchanged at all three experimental days (Fig. 3d–m), suggesting that signaling pathways activated by rhNGF can induce heat and mechanical hyperalgesia without sensitizing itch to pruritogens.

**Figure 4:**
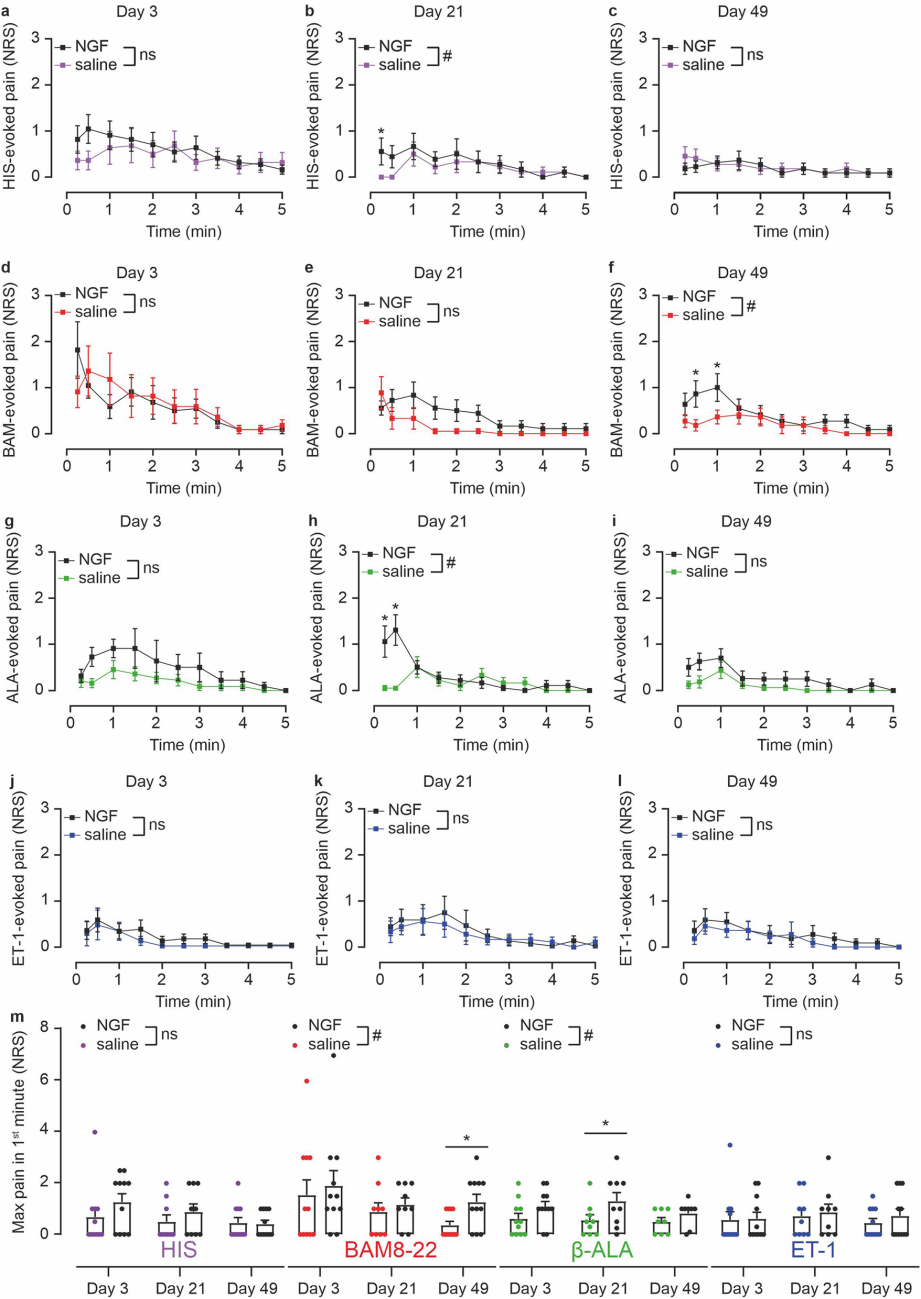
rhNGF enhances pain after injections of non-histaminergic pruritogens. (**a–l**) Pain ratings (NRS, 0–10) upon HIS (**a–c**), BAM8–22 (**d–f**), β-ALA (**g–i**), and ET-1 (**j–l**) recorded for 5 min at 3 days (n = 11), 21 days (n = 9) and 49 days (n = 11; n = 8 for β-ALA) post injection of rhNGF (black symbols) and saline (colored symbols), respectively. rhNGF treatment altered pruritogen-evoked pain for HIS and β-ALA at day 21 (p < 0.05) and for BAM8–22 at day 49 (p < 0.03; both marked with hash signs). Asterisks indicate significant differences at particular time-points (all p < 0.05) and data are depicted as mean ± standard error (SEM). (**m**) Maximum pain during the first minute after pruritogen delivery. In comparison to saline, rhNGF significantly enhanced BAM8–22- and β-ALA-induced pain (p < 0.02 and p < 0.01, marked by hash signs). In particular, pain was stronger at day 49 for BAM8–22 (p < 0.02) and at day 21 for β-ALA (p < 0.05; both marked by asterisks). Data are depicted as mean ± standard error (SEM) with overlay of individual data points.

In parallel to itch ratings, subjects were also asked to report on pain they experienced after pruritogen delivery. Increased pain ratings were observed at the rhNGF-sites only during the first minute post pruritogen injection (Fig. 4a–l). Quantification of the rhNGF effect during this period revealed that ET-1- and HIS-induced maximum and integrated pain were not changed by rhNGF (Fig. 4m, Fig. S2A+D). In contrast, pain ratings induced by BAM8–22 or β-ALA were enhanced at rhNGF-treated sites (Fig. 4m, Fig. S2B+C), in particular for BAM8–22 at day 49 (p < 0.02) and β-ALA at day 21 (p = 0.05).

**Figure 5:**
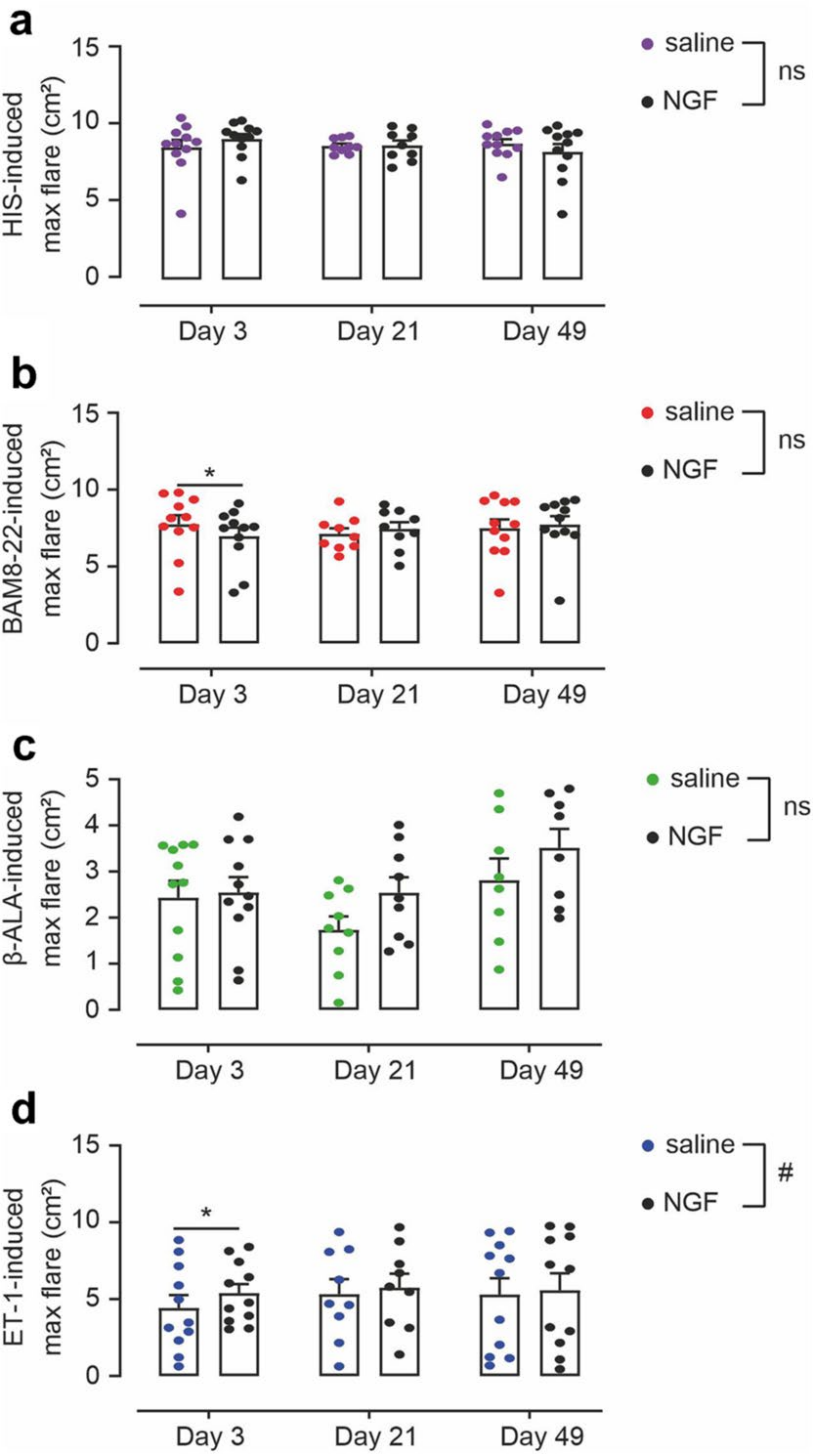
rhNGF has minor effects on pruritogen-induced flare responses. (**a–d**) Maximum flare responses extracted from a 5-min observation period after HIS iontophoresis (**a**) or after injections of BAM8–22 (**b**), β-ALA (**c**), and ET-1 (**d**) measured at 3 days, 21 days and 49 days post injection of rhNGF (black symbols) and saline (colored symbols), respectively. No effects of rhNGF were detected for HIS and β-ALA, respectively (n.s.). For BAM8–22, a slight rhNGF-induced flare decrease was detected at day 3 (p < 0.01, marked with asterisk), whereas ET-1-induced flare was enhanced by rhNGF (overall p < 0.03, marked by hash sign), in particular at day 3 post rhNGF (p < 0.05, marked with asterisk). Data are depicted as mean ± standard error (SEM) with overlay of individual data points.

As shown above, three of the four pruritogens used in our study induced significant flare reactions. We assessed whether pruritogen-induced flare reactions would be increased after rhNGF treatment. Maximum flare responses after HIS iontophoresis and β-ALA injections were not significantly different in rhNGF-treated skin (Fig. 5a+c). For BAM8–22 and ET-1, flare reactions differed significantly at day 3 post rhNGF treatment (Fig. 5b+d), but the effects were small. Furthermore, BAM8–22-induced flare reaction was diminished (− 10%; p < 0.01), while ET-1-evoked maximum flare was enhanced by rhNGF (+ 18%; p < 0.05). Quantifying integrated flare responses over the whole observation period of 5 min after pruritogen delivery gave similar results (Fig. S3).

**Figure 6:**
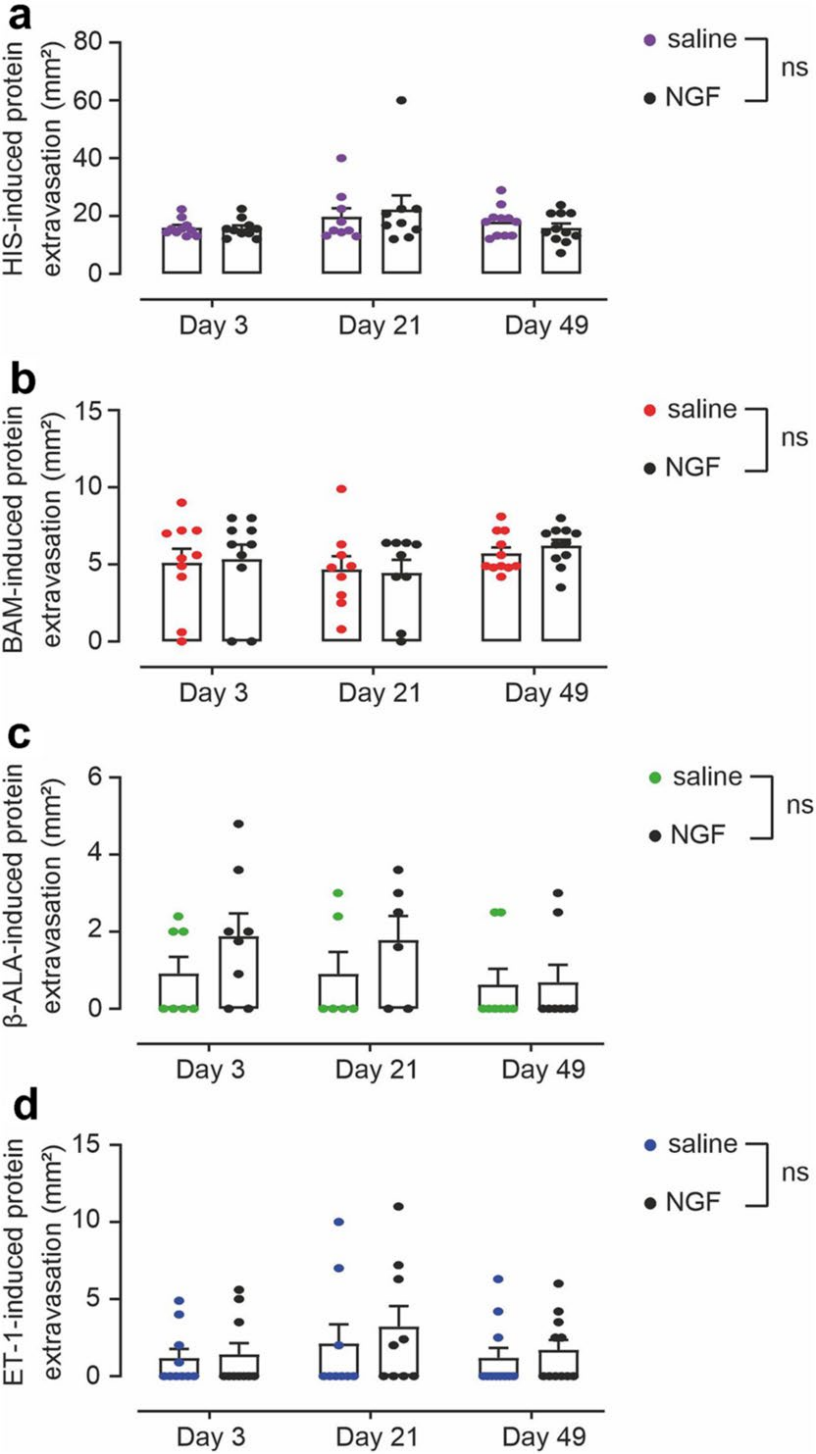
rhNGF does not impact pruritogen-induced protein extravasation. (**a–d**) Protein extravasation measured 5 min after iontophoretic delivery of HIS (**a**) or after injections of BAM8–22 (**b**), β-ALA (**c**), and ET-1 (**d**) at 3 days, 21 days and 49 days post injection of rhNGF (black symbols) and saline (colored symbols), respectively. No significant effects of rhNGF pretreatment were detected for any pruritogen at any experimental day (n.s.). Data are presented as mean ± standard error (SEM) with overlay of individual data points.

We further examined whether rhNGF treatment alters the protein extravasation (wheal) after pruritogen administration. HIS-induced protein extravasation as well as the minute wheal response upon BAM8–22 injection was not affected by rhNGF treatment (Fig. 6a+b). The same was true for ET-1 (Fig. 6d) as well as the response to β-ALA, which did not cause a protein extravasation under naïve conditions and that was not changed after rhNGF treatment either (Fig. 6c).

## Discussion

NGF via it`s high- and low-affinity receptors NTRK1 and NGFR is a well-established factor in nociceptive priming^32^ and a single rhNGF injection into the skin of healthy subjects induces hyperalgesia lasting several weeks^17,18,33^. In addition to this pro-algesic function, sensitizing effects of NGF in chronic itch have been suggested. Elevated NGF-levels were found in AD and psoriasis patients and correlated with the reported itch severity^26,34,35^ and increased skin innervation^36^. In fact, a phase 2B clinical trial revealed that itch in psoriasis patients could be successfully alleviated by NTRK1 inhibition^28^, furthering a causal link between upregulated NGF-NTRK1 interaction in the skin and chronic itch. Increased NGF-levels were also reported in skin biopsies from positive patch-tests, adding contact eczema as another inflammatory skin disease where itch and elevated NGF-levels in the skin coincide^27^. In the context of our present investigation, this is of particular significance since LaMotte and colleagues recently established an experimental human contact eczema model that was characterized by spontaneous itch and enhanced pruritus to BAM8–22, β-ALA and HIS^12^. In NGF-sensitized human skin, chemically induced itch was investigated previously showing that HIS-dependent itch is not altered by the pre-treatment^29,37^. In contrast, non-histaminergic itch induced by cowhage was elevated at day 21 after NGF challenge, i.e. when mechanical hypersensitivity was maximum^37^, but was unchanged during early heat hyperalgesia at day 3–4^29,37^. Herein, we therefore tested whether experimental NGF-induced skin sensitization of healthy volunteers produces a generalized sensitization to non-histaminergic pruritogens in parallel to thermal (day 3) or mechanical (day 21) hyperalgesia^18,38,39^.

### Pruritogen-evoked itch and flare responses

HIS delivery to the skin causes itch and a concomitant axon-reflex flare by activation of CMi-nociceptors^1,2^. In contrast, non-histaminergic pruritogens are thought to signal itch via distinct afferents, which is primarily based on results obtained with cowhage^40^. The cysteine protease mucunain was determined to be the itch-inducing active ingredient of cowhage spicules^41^. However, neuronal responses to mucunain with respect to the involved receptor proteins and afferent classes are rather complex^40–44^ and we therefore used BAM8–22, β-ALA and ET-1 as a set of better-defined non-histaminergic pruritogens. We detected flare reactions, one of the hallmarks of HIS-induced CMi activation, after injections of BAM8–22 and ET-1 that were only slightly smaller than the HIS-induced flare, whereas β-ALA did not induce such a reaction. This is in good agreement with previous results from our own and other groups that delivered these chemicals via intradermal microinjection^6,8,45–47^, but differs from studies that administered ET-1 superficially by iontophoresis or BAM8–22 focally into the skin using inactivated cowhage spicules^5,48^. The lack of a flare response in the latter studies might indicate that CMi activation and flare reaction after injection of ET-1 or BAM8–22 potentially constitute an indirect effect caused by a pruritogen-induced HIS release from skin mast cells as one plausible scenario. Previous studies indeed found expression of the BAM8–22 and ET-1 receptors MRGPRX1 and EDNRA on mast cells^49–52^, providing a structural substrate for this mechanism. ET-1 was already shown to induce an increase of skin HIS content, measured in microdialysis samples, but to a much lower extent in comparison to that induced by the mast cell activator codeine^6^. Additionally, we did not determine a substantial protein extravasation caused by BAM8–22 or ET-1 herein, which is, apart from the flare reaction, another strong indicator for HIS involvement. Thus, a partial depletion of HIS from skin mast cells by BAM8–22 and ET-1 achieved by injection of the compounds but not by superficial or focal delivery is the most likely scenario explaining our results. To further distinguish between HIS-dependent and -independent mechanisms of BAM8–22 and ET-1, we used the anti-histamine Cet and analyzed its effect on the pruritogen-induced itch and flare responses. ET-1-induced itch ratings and flare reactions were partially inhibited, which is in agreement with previous studies and indicates that ET-1 is not a *bona fide* non-histaminergic pruritogen^45,46,48^. For BAM8–22, only flare reactions were significantly reduced after Cet and thus depended on histamine receptor H1 (HRH1) activation while itch ratings were unaffected. Notably, after delivery of BAM8–22 by means of autoclaved cowhage spicules, the corresponding itch was likewise unaffected by an anti-histaminergic skin cream^5^. We therefore conclude that BAM8–22 exerts a direct effect on sensory afferents that produces itch. In addition, if BAM8–22 is injected and thus delivered deeper into the skin, a HIS-dependent flare develops that does not add to the direct BAM8–22-dependent itch. In agreement with this notion, we could not detect additive effects on itch when we co-injected supra-threshold amounts of BAM8–22 and HIS^8^. Collectively our study substantiates that BAM8–22, β-ALA and ET-1 induce itch in humans via HIS-independent (BAM8–22, β-ALA) or only partially HIS-independent (ET-1) mechanisms. However, we also provide evidence that “non-histaminergic pruritogens” can enact HIS-dependent processes that can contribute to the direct itch signal and the characteristic flare response evoked by these compounds.

### Pruritogen-evoked itch in NGF-sensitized skin

In the established NGF model of human skin sensitization, we investigated if BAM8–22-, β-ALA- or ET-1-induced itch sensations were altered following rhNGF injections. Our experiments were conducted at day 3 and day 21 after rhNGF challenge, time points previously shown to coincide with maximum heat or mechanical hyperalgesia, as well as at day 49 post rhNGF injection when hypersensitivity had largely resolved^18^. To exclude that we injected the pruritogens outside of the sensitized area, we used the rhNGF-induced pinch- and pinprick-hyperalgesia that is confined to the immediate vicinity of the rhNGF injection spot, develops within 2 days and remains detectable even at day 49^38^. Injection pain associated with pruritogen delivery was enhanced at the rhNGF skin sites confirming that injections were performed into the correct pretreated spot. We cannot rule out that mechanical hypersensitivity contributed to increased injection pain by the pruritogens. However, significantly higher injection pain for β-ALA as compared to ET-1 suggests a pruritogen-specific component. Itch responses elicited by the non-histaminergic pruritogens BAM8–22, β-ALA- or ET-1 were not sensitized at any time-point of investigation post rhNGF injection when compared to their administration into vehicle treated skin sites. In contrast, we previously found that non-histaminergic itch induced by the insertion of cowhage spicules into the epidermis was sensitized at day 21 but not at day 7 after rhNGF injection^37^. The Lerner group has shown in overexpression systems that mucunain, the active ingredient of cowhage, can activate the human protease-activated receptors 2 and 4 (PAR2 and PAR4) as well as members from the human MRGPRX family, MRGPRX1 and MRGPRX2^41,42^. Given that itch responses elicited by BAM8–22 were not sensitized by rhNGF in our study, it is tempting to speculate that MRGPRX1-independent mechanisms dominate the sensitized cowhage-evoked itch after NGF. If NGF sensitizes specific receptor proteins in the same cell, this could indicate that MRGPRX2 or PAR2/4 are the dominating receptor proteins targeted by the neurotrophin. In support of such a mechanism, PAR2 expression in sensory neurons was shown to increase in an NGF-dependent fashion in a murine model of tongue cancer^53^. However, other cowhage receptor proteins were not investigated in that study. Alternatively, MRGPRX1-negative but cowhage-responsive primary afferent units could be the primary target of NGF sensitization. Psychophysics and electrophysiological data recorded from humans and monkey show that cowhage-induced itch is mediated primarily through polymodal C- and mechanosensitive Aδ-fibers^40,43,44^. In humans and pigs, both of these fiber types are sensitized by rhNGF injection^54,55^. We could recently show in monkey that polymodal C-fibers are the class of afferents primarily activated by the MRGPRX1 agonist BAM8–22^8^. Therefore, Aδ-fibers might underlie sensitization of cowhage-induced itch, but it remains unclear whether dermal Aδ-fibers in humans do also express MRGPRX1 receptors as shown for human dental pulp nociceptors^56^. Considering that polymodal C-fibers are sensitized by NGF, it is additionally surprising that volunteers did not report increased itch after BAM8–22 injection. Apparently, molecular and neuronal pathways specific for BAM8–22-induced itch are not affected by NGF sensitization. In contrast, we found that pain sensations induced by injections of BAM8–22 and β-ALA were sensitized in rhNGF-treated skin, which supports previous data showing that rhNGF can sensitize chemically induced nociceptor responses in humans^15^. On the other hand, our results are surprising given that in induced human contact eczema elevated NGF levels and a sensitization of itch but not pain evoked by BAM8–22 and β-ALA were described^12,27^. Apparently, a single injection of NGF in non-inflamed skin is sufficient to sensitize for pain but not for itch induced by non-histaminergic pruritogens. In clinical non-histaminergic itch conditions associated with elevated NGF-levels and chronic inflammatory skin, the presence of inflammatory mediators (e.g. cytokines) may be relevant for enhanced itch in patients^57^. In healthy human subjects, an enhancement of non-histaminergic itch during acute NGF signaling may require additional inflammatory conditions at the time of stimulation. Indeed, the combination of UVB-inflammation with rhNGF had supra-additive effects on evoked pain in human skin^38,39^ and it would therefore be of interest to evaluate non-histaminergic itch under such experimental conditions. Another explanation for the opposing effects on itch and pain evoked by the same mediator in our NGF model compared to allergen- or SADBE-induced eczema^12,27^ are likely additional mediators present in eczematous skin, which however have not been identified yet. Finally, central mechanisms may account for a diverse sensory processing, for instance ongoing itch described in the contact eczema model^12^ might impact spinal circuitry, as shown in AD patients that perceived normally painful stimuli as itch^58^. Alternatively, NGF sensitization might primarily increase discharge frequency of nociceptors that is crucial to encode pain intensity with instantaneous discharge frequencies reaching 40 to 100 Hz^59^. In contrast, the peak frequencies in histamine responses of human pruriceptors are < 10 Hz^2^. Similarly, monkey and human polymodal nociceptors respond to cowhage or histamine with discharge frequencies below 10 Hz, much lower than their responses to mechanical stimulation (50–100 Hz)^8,60^. Thus, an assumed increase of discharge frequency following NGF^59^ might only translate into sensitization of pruritogen-induced pain, but not itch for which alternative encoding concepts are discussed^61^.

To sum up, our study demonstrates that a single injection of rhNGF is not sufficient to sensitize itch induced by the pruritogens BAM8–22, β-ALA, ET-1 and HIS in human skin. Permanently elevated NGF levels in an inflammatory context may be required for such sensitization, as present for instance under clinical conditions. However, increased pain responses post rhNGF to the pruritogens BAM8–22 and β-ALA indicate that human nociceptors expressing MRGPRX1 and MRGPRD^8^ appear particularly susceptible to NGF-dependent plastic changes, but these changes are not sufficient to sensitize their encoding of itch. Considering the correlation of increased NGF expression and itch severity in chronic inflammatory skin associated with pruritus^26,34,35^, our results do not rule out an important role of NGF in patients suffering from itch in these diseases.

## Methods

The study protocol and experimental procedure was approved by the Ethic Committee II, located at the Medical Faculty Mannheim of the University of Heidelberg. Experiments were conducted in accordance with the Declaration of Helsinki. 11 subjects (6 female, 5 male, average age 36 ± 4 years) were informed about design, aim and duration of the entire study, and signed written informed consent to participate.

### Study protocol

Subjects were familiarized with the experimental procedures and the use of a numeric rating scale (NRS) with the endpoints 0 (no itch or pain) and 10 (maximum itch or pain that can be imagined) to estimate the magnitude of the perceived sensation. The study protocol comprised two experiments, one for the recording of the somatosensory responses to the injection of pruritogens before and after intake of Cet, a HRH1-antagonist, and another for the longitudinal assessment of NGF-induced sensitization to injected pruritogens. Both sessions were investigated in the same subjects but with a time interval of 6 months in between.

### Pruritogens

The pruritogens BAM8–22, β-ALA, ET-1 and HIS were investigated. BAM8–22 was purchased from GenScript (custom synthesis services, Leiden, Netherlands) and all other compounds were supplied from Merck KGaA (Darmstadt, Germany). Concentrations of BAM8–22 (0.4 µg/µl), β-ALA (9 µg/µl), and ET-1 (1 µM) were prepared in sterile ECF (for composition see^10^). A 1% histamine di-hydrochloride solution was prepared in de-ionized water (Merck Millipore, Germany) and delivered into the skin by iontophoresis (1 mA for 20 s, equaling 20 mC) (World Precision Instruments, A360 Stimulus Isolator, Friedberg, Germany) to serve as positive pruritus control to BAM8–22, β-ALA, and ET-1, respectively. For iontophoresis, a silver-anode (diameter 5 mm) was equipped with a HIS-soaked cotton swap and as cathode a Kendall^®^ ECG electrode (diameter 24 mm, Covidien Medtronic, Meerbusch, Germany) was attached to the skin surface distally to the anode.

### Nerve growth factor

Recombinant human NGF (rhNGF, catalog number 130-093-971) lacking the two C-terminal amino acids of human β-NGF (UniProtKB—P01138) was purchased from Miltenyi Biotec (Bergisch-Gladbach, Germany) and dissolved in sterile 0.9% saline immediately prior to its injection. Volunteers received four 50 µl intradermal rhNGF injections (Becton Dickinson 30G syringe, Heidelberg, Germany) medial into their left and right volar forearm (2 injections per arm), 5 cm proximal to the cubital fossa and 5 cm distal to the wrist. Each injection contained 1 µg rhNGF. As vehicle control, two 50 µl intradermal 0.9% saline injections were placed equidistant between the two NGF injection spots in each forearm. All eight injection spots were labelled with a felt tip pen and marks copied on a transparent plastic wrap together with prominent moles, veins or birthmarks for later recognition of the rhNGF/vehicle sites. Somatosensory and vascular responses to injections with pruritogens or HIS iontophoresis (see below) of the rhNGF and vehicle pre-treated skin sites were performed on day 3, 21 and 49 after rhNGF/vehicle administration.

### Injection of pruritogens

Prior to pruritogen administration, we identified the rhNGF/vehicle spots using our transparent foil. rhNGF-sensitized skin sites were validated upon squeeze of a skin fold and an accompanied increased sensation reported by the volunteer. The exact injection site of the pruritogen in rhNGF-treated skin was determined by an elevated sensation reported by the subject upon 128 mN pinprick stimulation (MRC Systems GmbH, Heidelberg, Germany) delivered in comparison to an adjacent untreated site (subject eyes were closed throughout these tests) and each site was labeled on the skin with a felt tip pen. Thereafter, we injected intradermally 10 µl (Becton Dickinson U-100 Micro-Fine 30G insulin syringe, Heidelberg, Germany) of the pruritogens BAM8–22, β-ALA, and ET-1 in randomized order and random to the rhNGF and the contra-lateral vehicle pretreated site. Finally, following the injection of the pruritogens, we delivered iontophoretically HIS to a proximal rhNGF and the contra-lateral vehicle skin site (randomized order).

### HRH1-antagonist

An involvement of HRH1 in somatosensory responses evoked by the pruritogens was tested by orally administered 10 mg cetirizine di-hydrochloride (1 pill Zyrtec^®^, UCB Pharma GmbH, Monheim, Germany). Prior to medication, 10 µl of each pruritogen was injected intradermally into untreated volar forearm skin and the corresponding somatosensations and vascular responses recorded as described below. HIS was administered iontophoretically (1 mA for 20 s = 20 mC) into a proximal volar forearm skin site as positive control after injections of the pruritogens. Thereafter, each volunteer swallowed the Cet tablet with 50 ml tab-water and sensory as well as vascular assessments to pruritogen injection and HIS iontophoresis were repeated three hours later. Investigations exploring the impact of HRH1 were conducted six months after rhNGF skin challenge to ensure the absence of a potential neurotrophin sensitization.

### Somatosensation and vascular responses

#### NRS for itch and pain

Subjects were instructed to rate on the NRS (endpoints 0 and 10, see above) the maximum perceived pain during injection of the pruritogen. Immediately after pruritogen injection or HIS iontophoresis, respectively, intensity of both itch and pain were recorded separately in 15 s intervals for 30 s and thereafter in 30 s intervals for 5 min. The AUC as an integrated measure of the recorded sensation and the maximum sensation during the 5 min period were used for group comparisons.

#### Laser doppler imaging (LDI)

Widespread increase of skin blood flow (axon-reflex-mediated vasodilation) was monitored by LDI (Moor Instruments Ltd, LDI2, Axminster, UK). The device was mounted 50 cm perpendicular to the skin surface and the laser scan covered an area of 12.25 cm^2^ around the pruritogen administration site. Each imaging sequence comprised one baseline image of skin blood flow prior to and ten images recorded in 30 s intervals after administration of the pruritogens. Off-line analysis of the sequence was performed with dedicated software (MoorLDI, V5.0). On a per-pixel basis, a significant increase of skin blood flow was accepted if the perfusion (flux) exceeded the 95% percentile (mean + twofold SD) of the baseline image distribution. The flare area (cm^2^) of increased blood flow around the stimulation site was then calculated from the area of pixels with significant flux increase.

#### Protein extravasation

The wheal formation after substance application indicates an extravasation of plasma proteins from post-capillary venules into the surrounding tissue. We measured (in mm) the maximum diameter of the wheal along two orthogonal lines and across the stimulation site 5 min after pruritogen administration. The area of protein extravasation (mm^2^) was calculated by multiplication of the two diameters.

#### Statistics

Data were analyzed by repeated measurement analysis of variance (RMANOVA) and Bonferroni post-hoc test comparisons between the factorial groups “pruritogen”—“anti-histamine”—“rhNGF vs vehicle” using Prism 8.4.3 software (GraphPad Software, San Diego, USA). Due to a positive SARS-CoV-2 infection, two subjects had to be excluded from the sensitivity tests scheduled for day 21 after rhNGF injection. The correspondingly missing values were handled statistically by fitting a mixed effects model followed by Bonferroni post-hoc test (Prism 8.4.3). Values of p < 0.05 were accepted as significant difference between the factorial groups and are indicated in the figure legends with hash signs (mixed effects or RMANOVA) and asterisks (Bonferroni post-hoc test). Number of replicates, measures of center and variability and p-values are given in each figure legend and in the “Results” section.

## Supplementary Information

Supplementary Information.

## Data Availability

All data generated or analyzed during this study are included in this published article (and its Supplementary Information files) and are available from the corresponding author on reasonable request.
