# Publisher Correction: Microinjection of pruritogens in NGF-sensitized human skin

**DOI:** 10.1038/s41598-022-05811-w

**Published:** 2022-01-24

**Authors:** Hans Jürgen Solinski, Roman Rukwied, Martin Schmelz

**Affiliations:** Department of Experimental Pain Research, MCTN, Medical Faculty Mannheim, Heidelberg University, Theodor-Kutzer-Ufer 1-3, 68167 Mannheim, Germany

Correction to: *Scientific Reports* https://doi.org/10.1038/s41598-021-00935-x, published online 02 November 2021

In the original version of the Article, Figure 4 was a duplication of Figure 3. The original Article has been corrected.

