# Supplementary material for: Microinjection of pruritogens in NGF-sensitized human skin
Source: Sci Rep. 2021 Nov 2;11:21490. doi: 10.1038/s41598-021-00935-x (PMC8563721; doi:10.1038/s41598-021-00935-x)
Supplement: Supplementary file 1 — Supplementary Information. [file 41598_2021_935_MOESM1_ESM.docx]

**Microinjection of pruritogens in NGF-sensitized human skin**

Hans Jürgen Solinski^§^, Roman Rukwied^§,^*, Martin Schmelz

^§^ Authors contributed equally

* Corresponding author


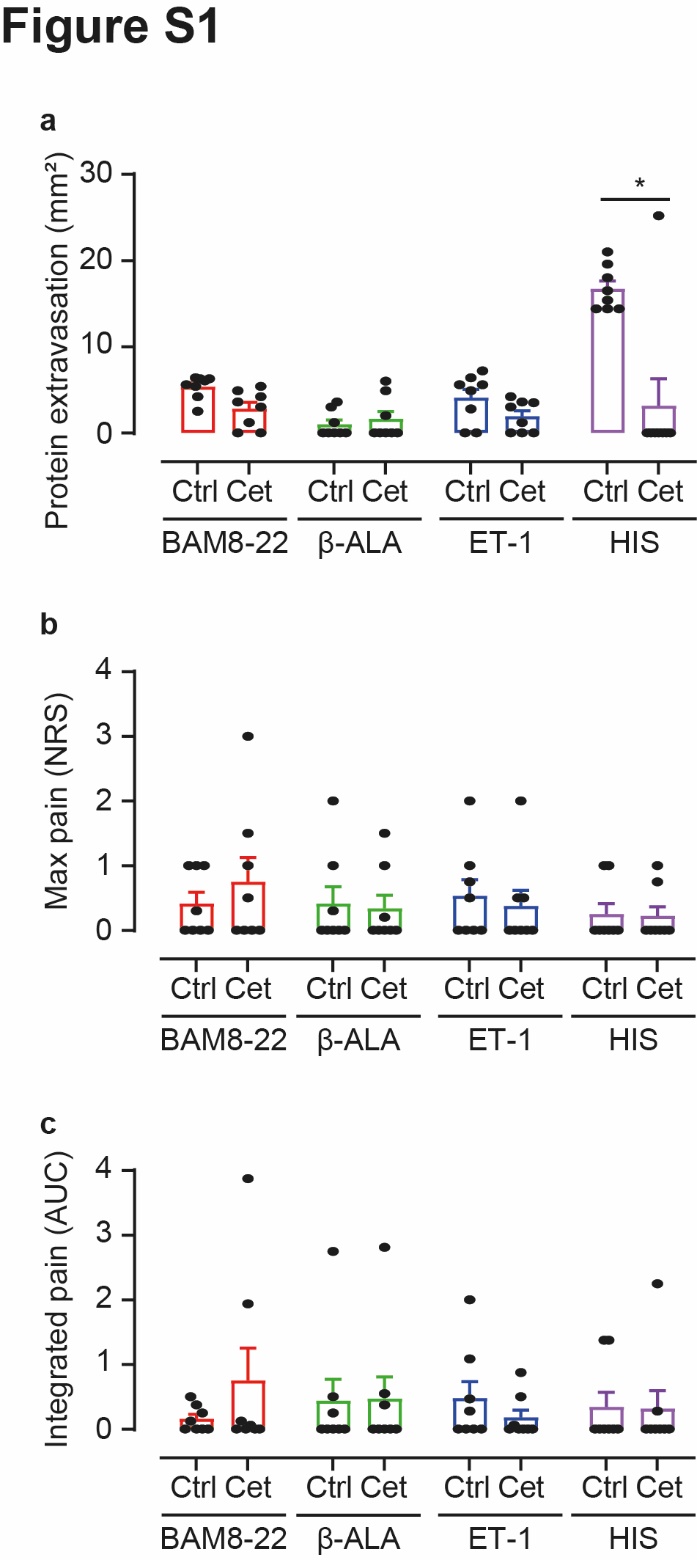


*Supplemental Figure 1 (to Fig. 2 of the manuscript): Protein extravasation and pain evoked by BAM8‑22, β-ALA and ET‑1 are independent of HIS.*

(A): Protein extravasation (mm^2^) to injections of BAM8‑22, β‑ALA, and ET‑1 or to iontophoretic delivery of HIS was measured before (Ctrl) and 3 hours after oral HRH1-antagonist cetirizine (Cet). Cet significantly inhibited HIS-induced protein extravasation (p < 0.0001, indicated by asterisk) but had no effect on the other pruritogens. Data are shown as mean ± standard error (SEM) with overlay of individual data points.

(B-C): Maximum pain (NRS) and integrated pain (AUC) during a 5-minute observation period after pruritogen delivery. Pruritogen-evoked pain was not altered by Cet (n.s.). Data are shown as mean ± standard error (SEM) with overlay of individual data points.


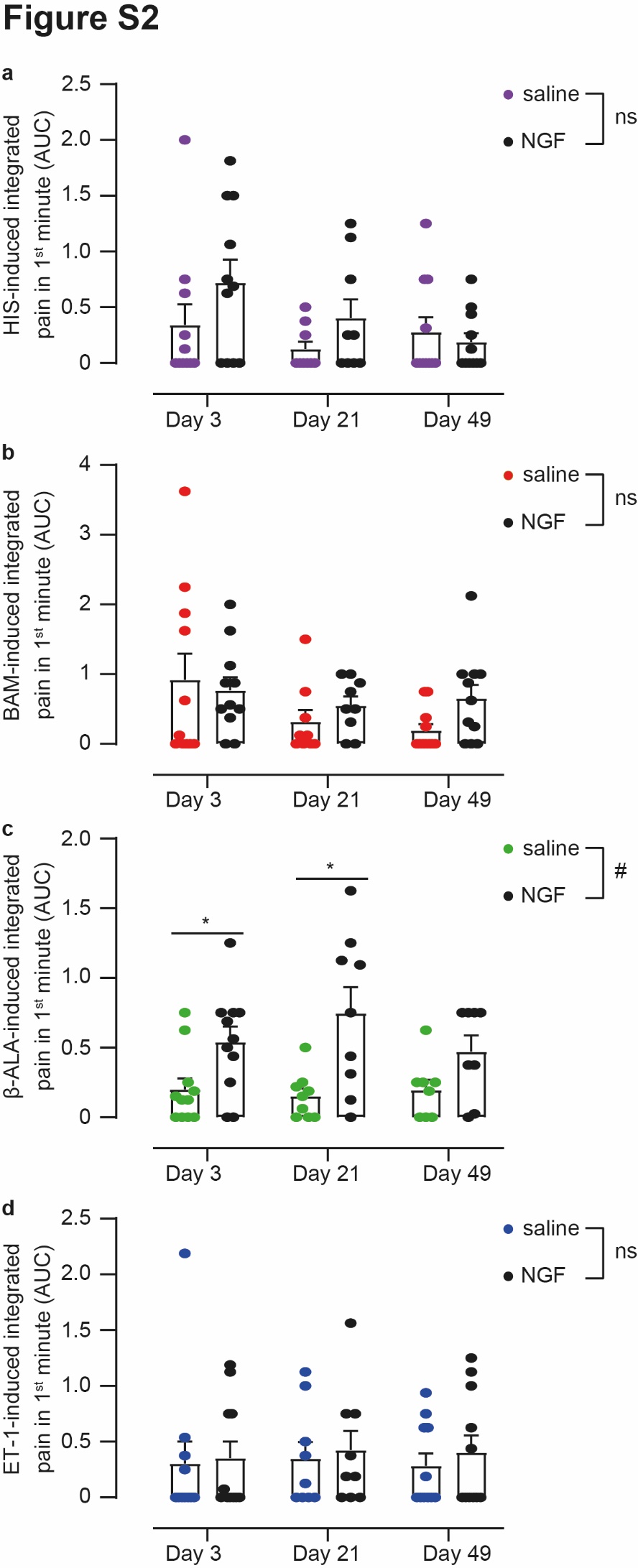


*Supplemental Figure 2 (to Fig. 4 of the manuscript): rhNGF enhances pain to injections of non-histaminergic pruritogens.*

(A-D): Integrated pain ratings (AUC) to iontophoretic delivery of HIS (A) or to injections of BAM8‑22 (B), β‑ALA (C), and ET‑1 (D) recorded at 3 days, 21 days and 49 days post injection of rhNGF (black symbols) or saline (colored symbols), respectively. rhNGF did neither enhance or diminish integrated pain to HIS, BAM8‑22 or ET‑1 at any experimental day (n.s.). However, β‑ALA-induced integrated pain was overall substantially enhanced by rhNGF (p < 0.0005, marked with hash sign), in particular at day 3 and 21 post rhNGF injection (both p < 0.05, marked with asterisks). Data are shown as mean ± standard error (SEM) with overlay of individual data points.


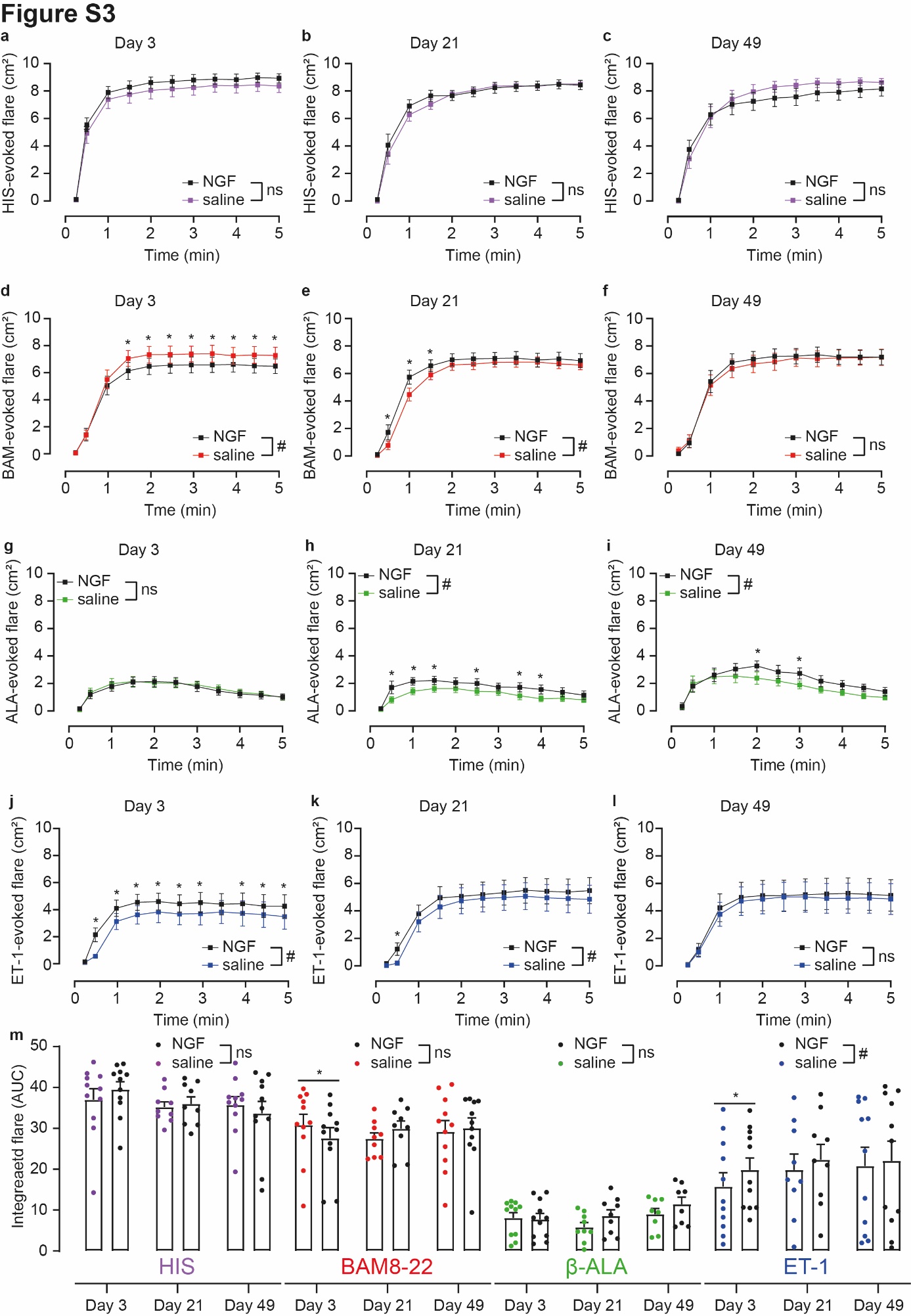


*Supplemental Figure 3 (to Fig. 5 of the manuscript): rhNGF has minor effects on pruritogen-induced flare responses.*

(A-L): Flare responses (cm^2^) recorded by laser Doppler imaging (LDI) to iontophoretic delivery of HIS (A-C) or to injections of BAM8‑22 (D-F), β‑ALA (G-I), or ET‑1 (J-L) recorded at 3 days (n = 11), 21 days (n = 9) and 49 days (n = 11; n = 8 for β‑ALA) post injection of rhNGF (black symbols) or saline (colored symbols), respectively. At specific experimental days rhNGF treatment altered pruritogen-evoked flare responses (all p < 0.05, marked with hash signs). These changes were driven by differences at specific time-points during the 5-minute LDI observation period (all p < 0.05, indicated with asterisks). Data are shown as mean ± standard error (SEM).

(M): Integrated flare responses (AUC) extracted from the 5-minute observation period after pruritogen delivery. No effects of rhNGF were detected for HIS and β‑ALA, respectively (n.s.). For BAM8‑22, a slight rhNGF-induced flare decrease at day 3 was detected (p < 0.05, marked with asterisk). In contrast, the ET‑1-induced flare was overall enhanced by rhNGF (p < 0.02; marked with hash sign), particularly at day 3 post rhNGF injection (p < 0.05, marked with asterisk). Data are depicted as mean ± standard error (SEM) with overlay of individual data points.
